# An Online Mammography Database with Biopsy Confirmed Types

**DOI:** 10.1038/s41597-023-02025-1

**Published:** 2023-03-07

**Authors:** Hongmin Cai, Jinhua Wang, Tingting Dan, Jiao Li, Zhihao Fan, Weiting Yi, Chunyan Cui, Xinhua Jiang, Li Li

**Affiliations:** 1 School of Computer Science and Engineering, South China University of Technology, Guangzhou, 510006 China; 2 Medical Imaging Center, Shenzhen Hospital, Southern Medical University, Shenzhen, 510515 China; 3 The Third of Clinical Medicine, Southern Medical University, Shenzhen, 510515 China; 4 Department of Medical Imaging, Collaborative Innovation Center for Cancer Medicine, State Key Laboratory of Oncology in South China, Sun Yat-sen University Cancer Center, Guangzhou, 510060 China

**Keywords:** Breast cancer, Prognostic markers

## Abstract

Breast carcinoma is the second largest cancer in the world among women. Early detection of breast cancer has been shown to increase the survival rate, thereby significantly increasing patients’ lifespan. Mammography, a noninvasive imaging tool with low cost, is widely used to diagnose breast disease at an early stage due to its high sensitivity. Although some public mammography datasets are useful, there is still a lack of open access datasets that expand beyond the white population as well as missing biopsy confirmation or with unknown molecular subtypes. To fill this gap, we build a database containing two online breast mammographies. The dataset named by Chinese Mammography Database (CMMD) contains 3712 mammographies involved 1775 patients, which is divided into two branches. The first dataset CMMD1 contains 1026 cases (2214 mammographies) with biopsy confirmed type of benign or malignant tumors. The second dataset CMMD2 includes 1498 mammographies for 749 patients with known molecular subtypes. Our database is constructed to enrich the diversity of mammography data and promote the development of relevant fields.

## Background & Summary

Breast carcinoma is one of the most commonly diagnosed cancer and the second leading cause of death from cancer in women^1^. The popularity of mammography uptake in breast carcinoma treatment has dramatically improved the 5-year survival rate of breast carcinoma since the 1980s^2^. Due to the sensitivity of mammography and the heterogeneity of breast cancer lesions, invasive methods such as biopsy, surgery is critical to confirm the benign and malignant tumors, and the molecular subtypes to optimize the type of treatment^3^.

Advances in both imaging and computer have synergistically lead to a rapid rise of the artificial intelligence (AI) for breast imaging in the following three tasks: (1) Computer-aided detection (CADe)^4–9^ aims at locating suspect lesions such as mass and microcalcification, leaving the classification to the radiologist; and (2) Computer-aided diagnosis (CADx)^10–14^ aims to characterize the suspicious region of lesion and/or estimate its probability of onset; and (3) Findings of predictive image-based biomarkers^15–18^ by applying the computational methods to mine the potential relationships between image representation and molecular subtype, including luminal A, luminal B, HER2 positive, and Triple-negative. Although mammography imaging is rapidly growing in the three areas, the promising results of radiomics approaches have not been widely used in daily clinical practice. Limited data sharing is an essential reason for reducing the development of radiomics strategies.

In investigating the CADe and CADx, there are several datasets^19–23^ that are publicly and freely available to authorized investigators. The datasets involve the Digital Database for Screening Mammography (DDSM), the Mammographic Imaging Analysis Society (MIAS) database, the Image Retrieval in Medical Application (IRMA) project, and the Curated Breast Imaging Subset of DDSM (CBIS-DDSM). Notwithstanding these public datasets are useful, there is still a lack of open access datasets that expand beyond the white population, which will enable researchers to verify previous findings and make the dataset more diverse. Furthermore, the biopsy confirmed results, such as immunohistochemical or molecular subtype, for most of the current datasets are missing. Therefore, an open-access database consisting of large samples with immunohistochemical type is valuable for researchers who are interested in this domain or who require an independent database for cross-validation. In this study, we built a database that contained two branches labeled by Chinese Mammography Database (i.e., CMMD1 and CMMD2) for allowing researchers to investigate the relationships among image features, pathological assessment, and tumor molecular subtypes. Specifically, CMMD1 including 1026 cases diagnosed with benign or malignant tumors were collated to promote the development of the CADx and CADe. While the CMMD2 included 749 cases, its purpose is to investigate the relationship between image features of invasive carcinoma and molecular subtypes. Note, the cases in CMMD2 have more complete immunohistochemical markers than CMMD1. Both datasets involved mammography images and clinical data such as age, and benign or malignant tumor. Currently, it is available for research through the International Data-sharing Initiative. Our free data sharing can hasten the clinical application of radiomics approaches. Table 1 lists the popular and publicly available databases in the field of mammography.

**Table 1:** Statistics of popular and publicly available databases in the field of mammography.

Database	Number of cases	Number of images	Molecular subtype	Image categories	Origin
MIAS^23^	161	322	No	benign, malignant, normal	UK
DDSM^30^	2620	10480	No	benign, malignant, normal	USA
LAPIMO^22^	320	1400	No	benign, malignant, normal	Brazil
INBreast^21^	115	410	No	benign, malignant, normal	Portugal
BCDR-DOX, BCDR-N01^20^	1010	3703	No	benign, malignant, normal	Portugal
TCGA^31^	69	88	No	—	USA
OPTIMAM^32^	173319	2889312	Yes	benign, malignant, normal	UK
CMMD1	1026	2214	No	benign, malignant	China
CMMD2	749	1498	Yes	malignant	China

## Methods

### Patient recruitment

**Fig. 1:**
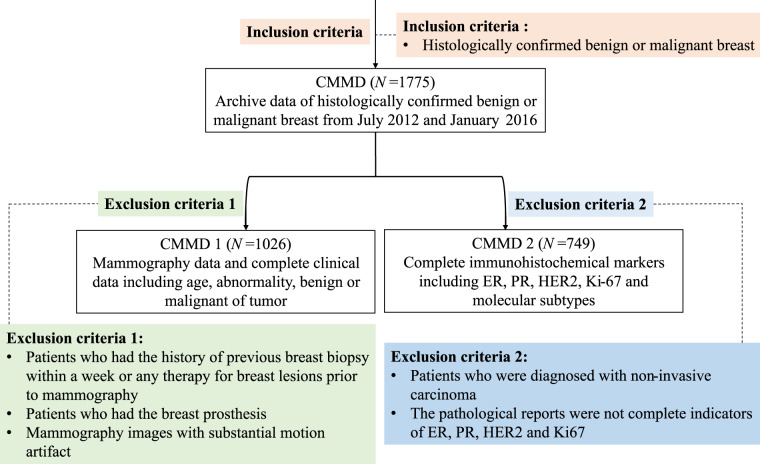
Recruitment pathway for patients in our study.

Ethical approval was acquired for this retrospective analysis, and the requirement to obtain informed consent was waived. Our study was conducted on 1775 patients (mean age: 47.56 year; range: 18–87 years) with benign or malignant breast who underwent mammography examination between July 2012 and January 2016. CMMD1 involves 1026 patients (mean age: 45.92 year; range: 17–84 years), which have the mammography data and complete clinical data. CMMD2 includes 749 patients (mean age: 49.82 year; range: 21–87 years) with complete immunohistochemical markers. Figure 1 illustrates the patient recruitment pathway, along with the inclusion and exclusion criteria. It is clear that CMMD1 and CMMD2 are the subsets of CMMD, CMMD1 merely distinguishes between benign and malignant patients (see the Exclusion criteria 1 in Fig. 1), while CMMD2 only contains malignant cases with detailed molecular subtypes (see the Exclusion criteria 2 in Fig. 1).

### Image collection and interpretation

Image data were acquired on a GE Senographe DS mammography system and a Siemens Mammomat Inspiration mammography system in the SunYat-sen University Cancer Center in Guangzhou, and the Nanhai Affiliated Hospital of Southern Medical University in Fushan, China. The scans were processed by the operator with a fixed operating procedure. For each subject, craniocaudal (CC) projection images and mediolateral oblique (MLO) projections images were obtained. In the released database, the raw images were stored as 8-bit grayscale in the Digital Imaging and Communications in Medicine format. All images were digitized at a resolution of 2294 × 1914 pixels.

Two radiologists with at least five years of experience performed mammography interpretation and guidance before surgery to determine which patients should be treated surgically. It was asked to refer to the standard readings of the breast imaging report and data system, established by the American College of Radiology^24^. By referring to commonly used X-ray classification methods, the images are divided into three types of masses, calcifications, and both. Note, the two radiologists independently reviewed the mammography in our study. When the results of the two doctors are inconsistent, they will combine the pathology report to further determine the type of abnormality.

### Pathological evaluation

In this study, biopsy samples were collected from all patients by core needle biopsy. The sample tissues were routinely stored as formalin-fixed and paraffin-embedded tissue blocks. The pathologist stained the section of biopsy tissue with hematoxylin and eosin (HE), analyzed the tissue morphology under the microscope. If necessary, surgery was performed to extract the suspicious lesion specimen. The immunohistochemistry test is conducted to determine the pathological result.

### Immunohistochemistry

According to the different expressions for immunohistochemistry including estrogen receptor (ER), progesterone receptor (PR), human epidermal growth factor receptor 2 (HER2), and Ki-67, invasive breast carcinoma is divided into four molecular subtypes, including Luminal A (ER+ and/or PR+, HEER2- and Ki-67 < 20%), Luminal B (ER+ and/or PR+ and Her2+ or Ki-67 > 20%), HER2-enriched (ER- and PR-, Her2+), and triple-negative (ER-, PR-, Her2-)^25^. The surgical specimens were fixed with 4% neutral buffer formaldehyde solution. The monoclonal antibodies were adopted for nuclear staining to evaluate the status of ER and PR. A negative test was defined as staining less than 1% (<1%) of tumor cells, while a positive test was defined as staining of greater than or equal to 1% (≥1%) of tumor cells. In assessing the expression of HER2, the specimen was first graded by IHC and scored by 0 to 3+, according to the recommendations of the American Society of Clinical Oncology/College of American Pathologists^26^. If there is no observed staining or faintly/barely perceptible membrane staining in less than 10% (<10%) of tumor cells, the score was set as 0. If there are greater than or equal to 10% (≥10%) of tumor cell membrane staining or the cell membrane staining faintly/barely noticeable, the score was marked as 1+. If there is weakly to moderately complete membrane staining observed in more than 10% (>10%) of tumor cells, the score was marked as 2+. In this case, the tissue was further evaluated by fluorescence *in situ* hybridization (FISH) analysis for HER2 gene amplification. In assessing the expression of Ki-67, immunostaining was performed by the monoclonal antibody Ki-67. The Ki-67 expression is divided between 0% and 100%. A cutoff value of 20% was used to classify the sample into low or high expression^27^.

**Fig. 2:**
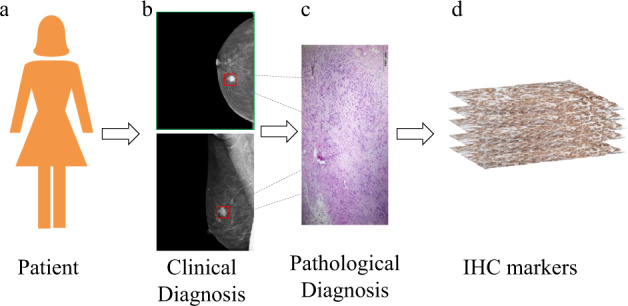
Study design for the construction of mammography data of breast. (**a**) Patients with lesions of breast were selected for the study. (**b**) MLO and CC molybdenum targets used as part of clinical diagnosis are shown in the scheme. (**c**) After the biopsy, resected tumors were routinely stored as formalin-fixed and paraffin-embedded (FFPE) tissue blocks and stained with hematoxylin and eosin (HE) for anatomic pathology. (**d**) Surgical specimens from surgery were evaluated by routine immunohistochemistry (IHC) to confirm the tumor of origin and molecular subtypes of each case.

To sum up, we list the clear and transparent about each step in the generation of the dataset, ultimately presenting a fully reproducible dataset, as shown in Fig. 2.

## Data Records

### Subject Identifiers

A unique identifier for each subject was identical in all two public datasets in this database. Subject IDs were 4-digit numbers in the form of D1-xxxx or D2-xxxx.

### Imaging and clinical data

**Fig. 3:**
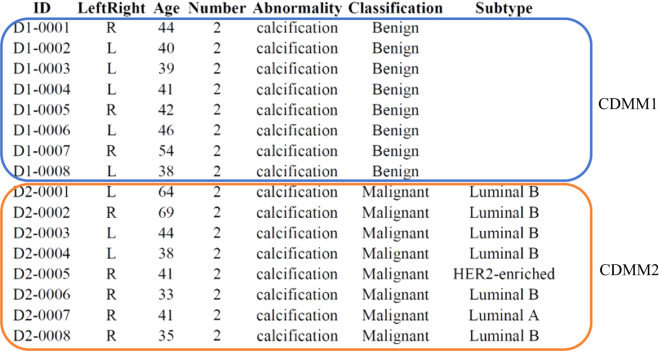
An example of clinical data for CMMD1 and CMMD2.

The CMMD collection^28^ contains breast mammography images and corresponding clinical data. Imaging, clinical data for all subjects are stored in The Cancer Imaging Archive https://www.cancerimagingarchive.net/ under 10.7937/tcia.eqde-4b16. Imaging data for all subjects are were store in the folder CMMD. All image data were processed using standard TCIA curation workflows. TCIA uses a standards-based approach for de-identification of images stored in the Digital Imaging and Communications in Medicine format. One comma-delimited file (CMMD_clinicaldata_revision.xlsx) contains clinical data for all subjects with unique subject identifiers. Table 2 lists the statistics on clinical-demographic of enrolled patients. Figure 3 is an illustrative example of clinical data for CMMD1 and CMMD2. As can be seen from the figure, the clinical data for CMMD1 contains age, image categories, and abnormality. Compared with CMMD1, CMMD2 further contains molecular subtypes that are able to assist the doctor for the clinical guidance or the related studies on immunohistochemistry.

**Table 2:** Statistics on clinical-demographic of enrolled patients.

		CMMD1	CMMD2
Number of cases		1026	749
Number of images		2214	1498
Age	Mean	45.92	49.82
	Median	45.00	49.00
Image categories	Benign	544	0
	Malignant	563	749
Abnormality	Mass	{726	417
	Calcifications	158	98
	Both	223	234
Molecular subtype	Luminal A	—	152
	Luminal B	—	376
	HER2-enriched	—	135
	Triple-negative	—	86

### Limitations of CMMD

Our data has some notable limitations. First, the sample size is not very large. Second, the ROI is not marked. We will add more available information and increase the amount of data in the future.

## Technical Validation

All data were collected by the hospital and used as part of the diagnosis, therefore all quality assurances were performed by the institution that collected the data.

## Usage Notes

The data of our previous publications^14,29^ are analyzed on CMMD1, while CMMD2 with molecular subtypes is our newly added data. All data are raw data without any preprocessing. We also welcome any cooperation with us to fully explore our dataset.

## Data Availability

Code for data cleaning and analysis is provided as part of the replication package. The code is uploaded to the Github platform: https://github.com/scutbioinformatics/CMMD.

## References

[1] National-Health-Service. Breast screening: professional guidance. https://www.gov.uk/government/collections/breast-screening-professional-guidance.

[2] Bi WL (2019). Artificial intelligence in cancer imaging: Clinical challenges and applications. CA: A Cancer Journal for Clinicians.

[3] Phi X-A, Tagliafico A, Houssami N, Greuter MJ, de Bock GH (2018). Digital breast tomosynthesis for breast cancer screening and diagnosis in women with dense breasts–a systematic review and meta-analysis. BMC cancer.

[4] Wang J, Yang Y (2018). A context-sensitive deep learning approach for microcalcification detection in mammograms. Pattern Recognition.

[5] Kooi T (2017). Large scale deep learning for computer aided detection of mammographic lesions. Medical Image Analysis.

[6] Samala RK (2016). Mass detection in digital breast tomosynthesis: Deep convolutional neural network with transfer learning from mammography. Medical Physics.

[7] Zhang, F. *et al*. Cascaded generative and discriminative learning for microcalcification detection in breast mammograms. In *Proceedings of the IEEE/CVF Conference on Computer Vision and Pattern Recognition*, 12578–12586, 10.1109/CVPR.2019.01286 (2019).

[8] Katzen J, Dodelzon K (2018). A review of computer aided detection in mammography. Clinical Imaging.

[9] Rodriguez-Ruiz A (2019). Stand-alone artificial intelligence for breast cancer detection in mammography: comparison with 101 radiologists. JNCI: Journal of the National Cancer Institute.

[10] Li J (2019). Predicting underestimation of ductal carcinoma *in situ*: a comparison between radiomics and conventional approaches. International Journal of Computer Assisted Radiology and Surgery.

[11] Agarwal R, Diaz O, Lladó X, Yap MH, Mart R (2019). Automatic mass detection in mammograms using deep convolutional neural networks. Journal of Medical Imaging.

[12] Arevalo J, Gonzalez FA, Ramospollan R, Oliveira JL, Lopez MAG (2016). Representation learning for mammography mass lesion classification with convolutional neural networks. Computer Methods and Programs in Biomedicine.

[13] McKinney SM (2020). International evaluation of an ai system for breast cancer screening. Nature.

[14] Cai H (2019). Breast microcalcification diagnosis using deep convolutional neural network from digital mammograms. Computational and Mathematical Methods in Medicine.

[15] Chen Y (2020). Evaluation of triple-negative breast cancer early detection via mammography screening and outcomes in african american and white american patients. JAMA Surgery.

[16] Ma W (2019). Breast cancer molecular subtype prediction by mammographic radiomic features. Academic Radiology.

[17] Hamidinekoo A, Denton E, Rampun A, Honnor K, Zwiggelaar R (2018). Deep learning in mammography and breast histology, an overview and future trends. Medical image analysis.

[18] Tagliafico AS, Piana M, Schenone D, Lai R, Houssami N (2019). Overview of radiomics in breast cancer diagnosis and prognostication. The Breast.

[19] Lee RS (2017). A curated mammography data set for use in computer-aided detection and diagnosis research. Scientific Data.

[20] Lopez MG (2012). Bcdr: a breast cancer digital repository. 15th International conference on experimental mechanics.

[21] Moreira IC (2012). Inbreast: toward a full-field digital mammographic database. Academic radiology.

[22] Matheus BRN, Schiabel H (2011). Online mammographic images database for development and comparison of cad schemes. Journal of digital imaging.

[23] Suckling, J. *et al*. Mammographic image analysis society (mias) database v1. 21, https://www.repository.cam.ac.uk/handle/1810/250394 (2015).

[24] Gard CC, Aiello Bowles EJ, Miglioretti DL, Taplin SH, Rutter CM (2015). Misclassification of breast imaging reporting and data system (bi-rads) mammographic density and implications for breast density reporting legislation. The breast journal.

[25] Yun SC, Pawlik TM, Vauthey JN (2017). 8th edition of the ajcc cancer staging manual: Pancreas and hepatobiliary cancers. Annals of Surgical Oncology.

[26] Wolff AC (2018). Human Epidermal Growth Factor Receptor 2 Testing in Breast Cancer: American Society of Clinical Oncology/College of American Pathologists Clinical Practice Guideline Focused Update. Archives of Pathology Laboratory Medicine.

[27] Bustreo S (2016). Optimal ki67 cut-off for luminal breast cancer prognostic evaluation: a large case series study with a long-term follow-up. Breast cancer research and treatment.

[28] Cui C (2022). The Cancer Imaging Archive.

[29] Wang J (2016). Discrimination of breast cancer with microcalcifications on mammography by deep learning. Scientific Reports.

[30] Bowyer K (1996). The digital database for screening mammography. Third international workshop on digital mammography.

[31] Clark K (2013). The cancer imaging archive (tcia): maintaining and operating a public information repository. Journal of digital imaging.

[32] Halling-Brown MD (2021). Optimam mammography image database: A large-scale resource of mammography images and clinical data. Radiology: Artificial Intelligence.

